# Utilizing Proteomic Approaches to Uncover the Neuroprotective Effects of ACE Inhibitors: Implications for Alzheimer’s Disease Treatment

**DOI:** 10.3390/molecules28165938

**Published:** 2023-08-08

**Authors:** Ming-Hui Yang, Tzu-Chuan Ho, Chin-Chuan Chang, Yuh-Shan Su, Cheng-Hui Yuan, Kuo-Pin Chuang, Yu-Chang Tyan

**Affiliations:** 1 Division of General & Digestive Surgery, Department of Surgery, Kaohsiung Medical University Hospital, Kaohsiung Medical University, Kaohsiung 807, Taiwan; 2 Department of Medical Laboratory Science and Biotechnology, Kaohsiung Medical University, Kaohsiung 807, Taiwan; 3 Department of Medical Imaging and Radiological Sciences, Kaohsiung Medical University, Kaohsiung 807, Taiwan; 4 Department of Nuclear Medicine, Kaohsiung Medical University Hospital, Kaohsiung 807, Taiwan; 5 School of Medicine, Kaohsiung Medical University, Kaohsiung 807, Taiwan; 6 Mass Spectrometry Laboratory, Department of Chemistry, National University of Singapore, Singapore 119077, Singapore; 7 International Degree Program in Animal Vaccine Technology, International College, National Pingtung University of Science and Technology, Pingtung 912, Taiwan; 8 Graduate Institute of Animal Vaccine Technology, College of Veterinary Medicine, National Pingtung University of Science and Technology, Pingtung 912, Taiwan; 9 Companion Animal Research Center, National Pingtung University of Science and Technology, Pingtung 912, Taiwan; 10 Graduate Institute of Medicine, College of Medicine, Kaohsiung Medical University, Kaohsiung 807, Taiwan; 11 Department of Medical Research, Kaohsiung Medical University Hospital, Kaohsiung 807, Taiwan; 12 Center for Tropical Medicine and Infectious Disease Research, Kaohsiung Medical University, Kaohsiung 807, Taiwan

**Keywords:** angiotensin-converting enzyme inhibitors, Alzheimer’s disease, proteomics, neuroblastoma

## Abstract

Two types of angiotensin-converting enzyme (ACE) inhibitors, lisinopril and benazepril HCl, were tested in neuroblastoma cells and found to upregulate low-density lipoprotein-receptor-related protein 1B (LRP1B) and 14-3-3 protein zeta/delta. Additionally, benazepril HCl was found to increase the expression of calreticulin. The upregulation of these proteins by ACE inhibitors may contribute to the amelioration of cognitive deficits in Alzheimer’s disease/dementia, as well as the clinically observed deceleration of functional decline in Alzheimer’s patients. This discovery suggests that the supplementation of ACE inhibitors may promote neuronal cell survival independently of their antihypertensive effect. Overall, these findings indicate that ACE inhibitors may be a promising avenue for developing effective treatments for Alzheimer’s disease.

## 1. Introduction

Neurodegenerative diseases, including Alzheimer’s disease, represent health challenges worldwide. If no helpful prevention strategies or effective therapeutic treatments are established, there may be more than 150 million patients in 2050 [1]. The current prevention strategies mainly focus on dietary intervention, such as increasing uptake of food that is rich in fatty acids (Omega-3 or 6) or docosahexaenoic acid (DHA). As for treatments, four drugs (donepezil, memantine, galantamine, rivastigmine) are approved belonging to two families: anticholinesterase inhibitors and anti-glutaminergics [2]. Nevertheless, these symptom-based treatments are not satisfying. Central-acting angiotensin-converting enzyme (ACE) inhibitors have been reported to prevent cognitive impairment [3,4] and decrease β-amyloid-dependent neurodegeneration [5] in animal models of Alzheimer’s disease. Clinical observations have also suggested that ACE inhibitors reduce the rate of functional decline in patients with Alzheimer’s disease and/or dementia [6,7,8,9,10,11]. Therefore, the inhibition of ACE activity was suggested to benefit patients with Alzheimer’s disease and/or dementia.

Unexpectedly, ACE was found to have hydrolytic activity capable of degrading β-amyloid [12,13,14]. Furthermore, overexpression of catalytically active ACE has been shown to reduce the burden of neurotoxic β-amyloid and preserve cognitive function in mice [15,16]. It was also suggested that ACE may be protective since elevated ACE levels reduces the risk of global brain atrophy and Alzheimer’s disease [17,18,19]. Controversially, these findings imply that the inhibition of ACE abolishes ACE-dependent clearance of β-amyloid, and hence the cognitive decline may be worsened.

Moreover, it was suggested that the ACE-mediated control of the renin-angiotensin system may play a role in the cognitive decline of patients with Alzheimer’s disease [6]. However, recent in vivo studies suggested that the amelioration of cognitive impairment achieved by perindopril, a centrally acting ACE inhibitor, was independent of its antihypertensive effect [3,20]. This finding strongly suggests that there are novel effective factors other than ACE that directly respond to ACE inhibitors and protect neuronal cells.

To solve the puzzle of whether supplementation with ACE inhibitors can benefit patients with Alzheimer’s disease and/or dementia, additional insights into ACE inhibitor-mediated gene regulation are indispensable. In this study, we explored specific proteins upregulated by ACE inhibitors using a differential proteomic approach. We were particularly interested in finding ACE-inhibitor-regulated proteins that potentially play functional roles in the protection of neuronal cells.

## 2. Results

### 2.1. Protein Identification by Mass Spectrometry

In order to identify the candidate proteins that may be related to ACE inhibitor treatments, each sample was analyzed by nano-HPLC-ESI-MS/MS in triplicate. After proteomic mass spectrometry analysis and a search through the Swiss-Prot database, a total of 77,219 peptides were identified across three groups, of which 25,368 were matched to 898 proteins. Subsequently, the most reliable proteins were selected based on the identification of at least three peptides each in the Mascot software (Version 2.2.1). There were 123, 159 and 121 proteins identified from the control, lisinopril-treated and benazepril-hydrochloride-treated groups, respectively. The number of upregulated proteins was 59 in the lisinopril-treated group and 55 in the Bbenazepril-hydrochloride-treated group. Of these, 41 proteins were identified in both treatments. The functions of these proteins were related to their association with the nervous system. A few proteins were identified to be more commonly up-regulated in ACE-inhibitor-treated neuroblastoma cells compared with nontreated cells. Ultimately, three proteins associated with Alzheimer’s disease were selected: 14-3-3 protein zeta/delta, low-density lipoprotein-receptor-related protein 1B (LRP1B), and calreticulin (Table 1 and Table 2).

**Figure 1:**
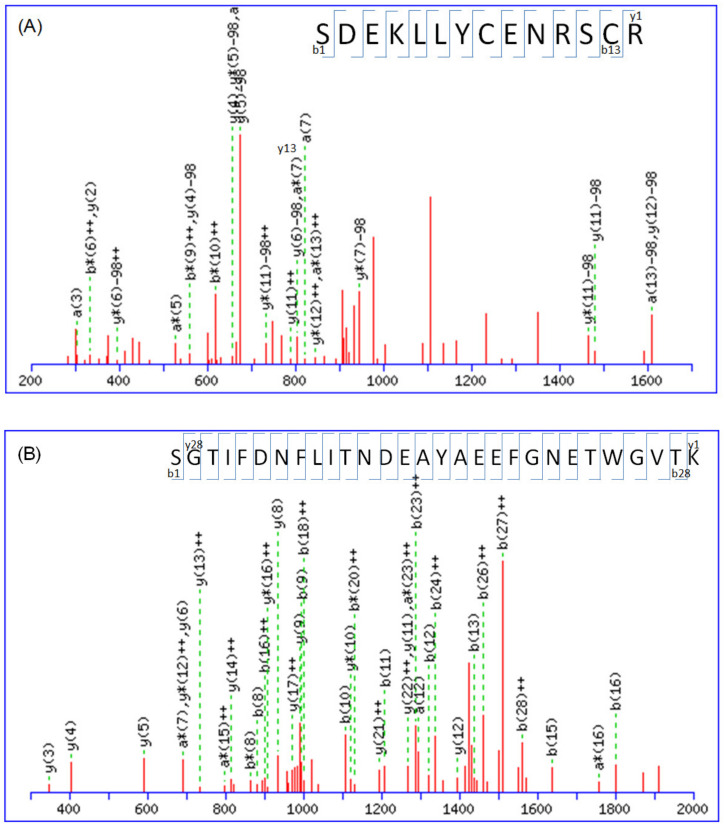

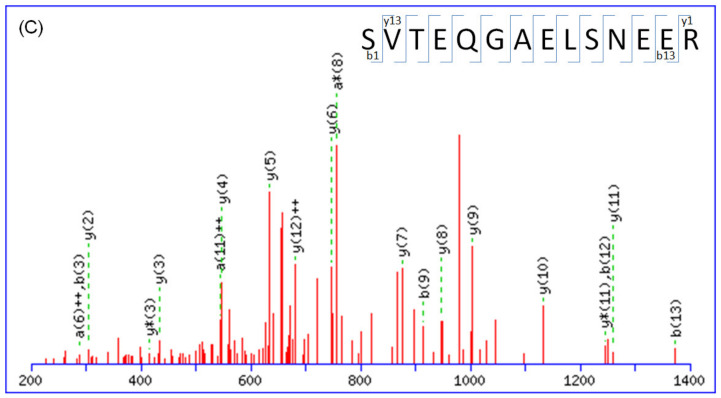
MS/MS spectral data of peptides obtained from ACE-inhibitor-treated neuroblastoma cells are shown (X axis: *m*/*z*; Y axis: intensity). The tryptic peptides have the following amino acid sequences: (**A**) K.SDEKLLYCENRSCR.R (*m*/*z* = 955.88, +2, from LRP1B), (**B**) K.SGTIFDNFLITNDEAYAEEFGNETWGVTK.A (*m*/*z* = 1090.16, +3, from calreticulin), and (**C**) K.SVTEQGAELSNEER.N (*m*/*z* = 774.85, +2, from 14-3-3 protein zeta/delta). The peptide sequences can be determined by interpreting the complete y-ion and b-ion series, as shown.

The fragmentation spectra obtained from the nano-HPLC-ESI-MS/MS analysis were searched against a nonredundant protein database using MASCOT. Proteins were considered present in the sample if they were identified by three or more unique peptides with MASCOT scores. No visual assessment of the spectra was performed; however, all MASCOT results were manually confirmed by assessing the overall quality of the MS/MS spectra. Figure 1 displays the typical MS/MS spectra of the identified peptides, which are tryptic peptides with *m*/*z* of 955.88 (doubly charged), 1090.16 (triply charged), and 774.85 (doubly charged). These peptides, with amino acid sequences of K.SDEKLLYCENRSCR.R, K.SGTIFDNFLITNDEAYAEEFGNETWGVTK.A, and K.SVTEQGAELSNEER.N, which originated from LRP1B, calreticulin, and 14-3-3 protein zeta/delta, respectively. The complete y-ion and b-ion series were interpreted to provide the peptide sequence.

**Figure 2:**
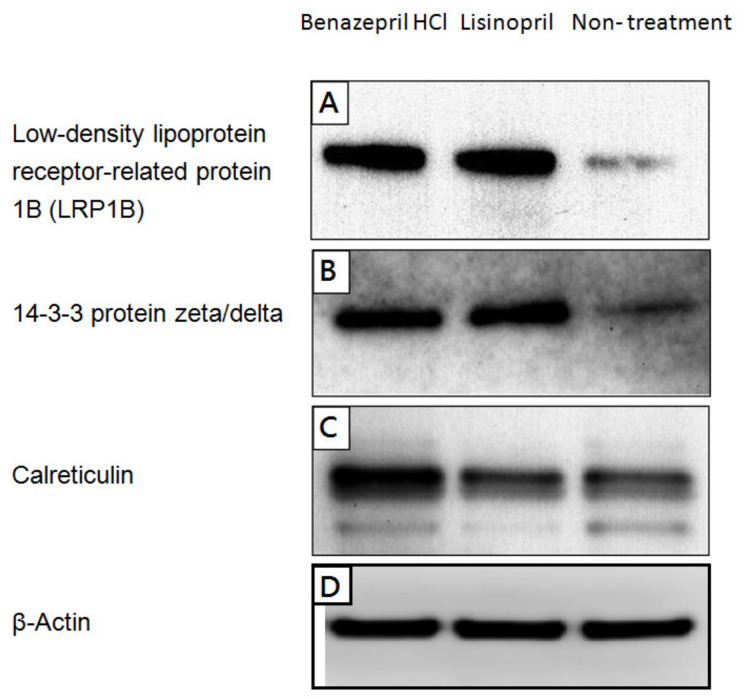
Cells were treated with 20 uM of lisinopril or benazepril HCl for 48 h and then harvested for protein. Protein expression levels of (**A**) low-density lipoprotein-receptor-related protein 1B (LRP1B) (**B**) 14-3-3 protein zeta/delta (**C**) calreticulin and (**D**) β-actin were detected by western blotting. β-actin was used as a loading control. Western blotting was performed in triplicate, and each presented image is a representative image from one of the replicates.

The differential expression levels of these proteins were verified by western blotting (Figure 2). These selected proteins, which were upregulated in both ACE-inhibitor-treated groups, play important roles in the nervous system and are also associated with Alzheimer’s disease. Both LRP1B and 14-3-3 protein zeta/delta were found to be upregulated in cells treated with either ACE inhibitor (Figure 2A,B), while calreticulin was predominantly upregulated by benazepril HCl (Figure 2C).

### 2.2. Alteration of Cellular Neuropathogenic Proteins by ACE Inhibitors

After treating cells with 1–100 μM of lisinopril or benazepril HCl for 48 h, the survival rates were between 120 and 250%. The survival rates were highest when cells were treated with 20 μM of lisinopril or benazepril HCl. No significant toxicity was observed in SH-SY5Y neuroblastoma cells treated with lisinopril or benazepril HCl at the concentrations used in each treatment.

**Figure 3:**
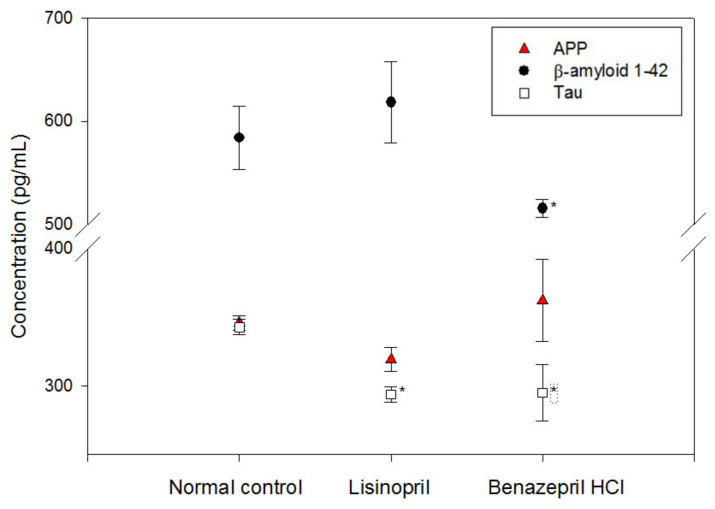
Confirmation of amyloid protein precursor (APP) expression in neuroblastoma cells showed slight but insignificant variation compared to the control group. In addition, the concentration of β-amyloid 1-42 showed a slight decrease after the addition of benazepril HCl. Furthermore, the concentration of total tau significantly decreased compared to the control group (mean ± standard error, n = 6, * *p* < 0.05, *t*-test).

The concentrations of amyloid protein precursor (APP), β-amyloid, and tau proteins in neuroblastoma cells were monitored (Figure 3). It was found that the APP concentration did not show any significant changes after treatment with ACE inhibitors, while the composition of β-amyloid seemed to be slightly affected by treatment with benazepril HCl. On the other hand, the concentration of tau protein was slightly downregulated by ACE inhibitor treatment.

**Figure 4:**
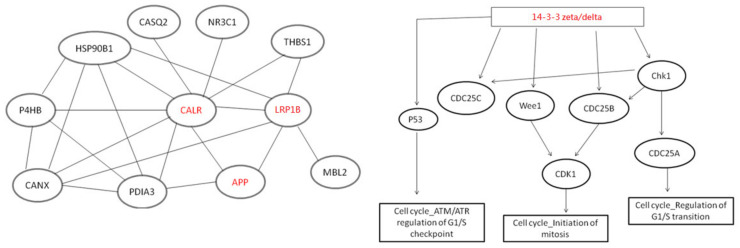
The protein–protein interaction pathways were generated using String v9.1 software. These pathways were then illustrated to demonstrate the interconnections among the identified proteins from the String Web software pathway analysis, including calreticulin (CALR), low-density lipoprotein-receptor-related protein 1B (LRP1B), amyloid protein precursor (APP), and 14-3-3 protein zeta/delta, among others.

In this study, by using String Web v9.1 software pathway analysis, several proteins were found to be involved in the related pathway. Although this was not shown in our wet lab work, the literature-based evidence constituted results from experiments such as western blot, immunohistochemistry, real-time PCR, proteomics, etc. The protein–protein interactions among these proteins are shown in Figure 4, where they exhibit complex interactions with other proteins.

## 3. Discussion

The neuroblastoma cell line model can be a valuable tool in research and drug development for several reasons. It provides a simplified yet representative system for studying Alzheimer’s disease (AD) and dementia. SH-SY5Y cells, which are tumor cells derived from neuroblastoma, stably express amyloid precursor protein and exhibit multipotent sympathetic function. These cells possess important neuronal biochemical and functional characteristics, including the expression of dopamine beta hydroxylase and the ability to convert glutamates into neurotransmitters. Consequently, SH-SY5Y cells are frequently utilized as a neuron model in neural research, encompassing studies on nerve cell differentiation, metabolism, neuropathy, and neuroprotective effects [21,22]. Moreover, this model enables researchers to evaluate the efficacy of potential therapeutic agents against various diseases. By exposing cells to testing compounds, effects on cell viability, proliferation or other parameters can be evaluated. Furthermore, the expression patterns of genes, proteins, and other biomolecules in these cells may reveal potential biomarkers with diagnostic or prognostic value in neuroblastoma patients.

### 3.1. Potential Neuroprotective Effect Mediated by ACE Inhibitors

LRP1B is a multifunctional endocytic receptor that participates in β-amyloid-peptide clearance [23]. LRP1B and related low-density-lipoprotein (LDL) receptors have been shown to inhibit APP endocytosis and reduce β-amyloid production [23,24]. LRP1B was recently identified as an essential mediator of C1q-induced protection against β-amyloid neurotoxicity in Alzheimer’s disease mouse models [25], which partly explains how ACE inhibitors reduce β-amyloid-dependent neurodegeneration [26]. LRP1B has been detected in neurons and astrocytes, and its ligands, including ApoE, α2-macroglobulin, and APP, have been found in the plaques of Alzheimer’s patients, playing a role in clearing β-amyloid and ApoE/α2-macroglobulin complexes in cell models [23,24]. LRP1B also plays a role in removing β-amyloid across the blood–brain barrier [26], and its reduced or absent expression may result in decreased clearance of β-amyloid and ApoE/α2-macroglobulin complexes, thereby increasing the risk of Alzheimer’s disease. Furthermore, studies have directly pointed out that the LRP1B protein reduces the internalization of APP cells and decreases β-amyloid production [23]. The LRP1B C/C genotype has been associated with a higher risk of Alzheimer’s disease [27].

The 14-3-3 protein family is a diverse group that plays a crucial role in regulating various cellular functions. One of its subtypes, 14-3-3 protein zeta/delta, interacts with other proteins, which affects cell cycle regulation, cell proliferation, apoptosis, transcription, and regulation of the cytoskeleton. Additionally, 14-3-3 protein zeta stimulates the phosphorylation of the tau protein, which is related to neurofibrillary tangles. Moreover, the 14-3-3 protein zeta/delta has been found to be upregulated in cells treated with lisinopril and benazepril HCl, and its neuroprotective functions are of particular interest [28]. These functions include manipulating the phosphorylation of tau and suppressing its aggregation [29,30,31]. Furthermore, 14-3-3 proteins interact with serine-arginine protein kinase 2 (SRPK2) and prevent the formation of hyperphosphorylated tau in Alzheimer’s disease [32,33]. These neuroprotective functions may partially account for the improvement in cognitive deficits resulting from ACE inhibitor supplementation. In summary, the 14-3-3 protein zeta/delta plays a vital role in regulating various signal transduction pathways that regulate cell cycle, neuron development, the cytoskeleton, and cell proliferation, and it has important implications in neuroprotective therapy.

Calreticulin, a 55–60 kDa protein found in the cytoplasm and nucleolus of neuronal cells and around the nuclei of neuroglial cells, is a binding partner of APP and LRP1, potentially playing a role in the clearance of β-amyloid [34,35]. Moreover, recent research has suggested that calreticulin functions as a negative biomarker of Alzheimer’s disease, indicating its positive role in preventing the disease [36]. Previous studies have shown that the expression of calreticulin mRNA and protein in microglial cells in the white matter of Alzheimer’s-disease patients is significantly lower than that of healthy individuals, leading to an increase in oxidative stress and mitochondrial damage, which in turn causes the production of β-amyloid in nerve endings [34,35]. Our experimental results demonstrate that the use of ACE inhibitors, such as benazepril HCl, can promote the expression of calreticulin in neurons, thereby reducing the expression of β-amyloid.

The results of this study showed that lisinopril and/or benazepril HCl upregulated LRP1B, 14-3-3 proteins, and calreticulin. These findings suggest that ACE inhibitors may have a neuroprotective effect against β-amyloid neurotoxicity and promote neuronal cell survival in Alzheimer’s disease and/or dementia by upregulating LRP1B, 14-3-3 protein zeta/delta, and calreticulin.

### 3.2. The role of ACE in ACE-Inhibitor-Mediated Amelioration of Cognitive Deficits

Previous studies showed that inhibition of ACE activity by ACE inhibitors did not increase β-amyloid accumulation in vivo [17,37,38], although ACE has been shown to have β-amyloid hydrolytic activity in vitro [12,14,39]. The role of ACE in the brain as a β-amyloid cleaner is uncertain. Additionally, it has been shown that the amelioration of cognitive deficits mediated by ACE inhibitors in Alzheimer’s disease is independent of the antihypertensive effect [3,20]. Metastudies by different research groups could not conclude whether ACE insertion/deletion polymorphism has a significant impact on Alzheimer’s disease patients, suggesting that ACE might play a minor role in Alzheimer’s disease [40,41,42]. Interestingly, ACE was not detectable in SH-SY5Y cells (unpublished data). Thus, the lisinopril/Benazepril-HCl-mediated regulation found in neuroblastoma cells was predominantly independent of the ACE protein.

To understand how ACE inhibitors trigger specific neuroprotective effects independent of ACE, further studies are essential. Prospective studies should focus on the molecular basis of ACE-inhibitor-triggered regulation in vivo. On the other hand, studying the (dis)similarity between the molecular interplay induced by ACE inhibitors and other renin-angiotensin-system-acting drugs, such as angiotensin-converting enzyme inhibitors and angiotensin II receptor blockers, is critical for understanding whether these drugs directly benefit Alzheimer’s disease/dementia patients regardless of hypertension. This knowledge is of great clinical importance in managing Alzheimer’s disease, dementia, and hypertension.

## 4. Materials and Methods

### 4.1. Cell Culture of SH-SY5Y Neuroblastoma Cell Line

Cell line SH-SY5Y (ATCC^®^ CRL-2266™, Bioresource Collection and Research Center, Hsinchu, Taiwan), a subline of the neuroblastoma cell line SK-N-SH, was purchased from ATCC Food Industry Research and Development Institute (Taipei, Taiwan). Cells were cultured (1 × 10^4^ cells/dish) according to the supplier’s recommendation and maintained in Dulbecco’s Modified Eagle Medium, Nutrient Mixture F-12 (DMEM/F12) with 10% fetal bovine serum (FBS) at 95% relative humidity (RH), 5% CO_2_, and 37 °C.

### 4.2. Treatment of ACE Inhibitors

Approximately 2.7 × 10^5^ cells were cultured in a 15 cm dish for 24 h, followed by treatment with 20 μM lisinopril or benazepril hydrochloride (HCl) for 48 h. The control groups were cultured without ACE inhibitors.

### 4.3. Protein Identification

Cultured cells were collected and lysed with 1 mM PMSF cell lysis buffer at −80 °C for 24 h. The cell lysate samples (100 μL) were then reduced, alkylated, and digested with trypsin at 37 °C for 12 h. Formic acid (2 μL) was added to each sample prior to mass spectrometric analysis for protein identification. Complex peptide mixtures were separated using RP-nano-HPLC-ESI-MS/MS (nano ACQUITY UPLC, Waters, Milford, MA, USA) coupled to an ion-trap mass spectrometer (LTQ Orbitrap Discovery Hybrid FTMS, Thermo, San Jose, CA, USA). The protein tryptic digests were fractionated at a flow rate of 400 nL/min with a nano-UPLC system (nanoACQUITY UPLC, Waters, Milford, MA, USA) coupled to an ion-trap mass spectrometer (LTQ Orbitrap Discovery Hybrid FTMS, Thermo, San Jose, CA, USA) equipped with an electrospray ionization source. For reverse-phase nano-UPLC-ESI-MS/MS analyses, a sample (2 μL) of the desired peptide digest was loaded into the trapping column (Symmetry C18, 5 μm, 180 μm × 20 mm) with an autosampler. Reverse-phase separation was performed using a linear acetonitrile gradient from 99% buffer A (100% D.I. water/0.1% formic acid) to 85% buffer B (100% acetonitrile/0.1% formic acid) in 100 min using the micropump at a flow rate of approximately 400 nL/min. Separation was performed with a C18 microcapillary column (BEH C18, 1.7 μm, 75 μm × 100 mm) using a nanoseparation system. As peptides were eluted from the microcapillary column, they were electrosprayed into the ESI-MS/MS by the application of a distal 2.1 kV spraying voltage with heated capillary temperature of 200 °C. Each cycle of one full-scan mass spectrum (*m*/*z* 400–2000) was followed by four data-dependent tandem mass spectra, with collision energy set at 35%. Fragmented peptides were analyzed with Mascot software (Version 2.2.1, Matrix Science, London, UK) and mapped to the Swiss-Prot human proteome database.

For proteolytic cleavage, only tryptic cleavage after arginine and lysine was allowed, and the maximum number of internal (missed) cleavage sites was set to two. Modifications of cysteine with carboxymethylation and methionine with oxidation were permitted. The mass tolerance for precursor peptide ions was set to 1.0 Da, and that for fragment ions was set to zero. Most differentially expressed proteins were then identified according to their Mascot score and coverage percentage. In this study, all protein identification results were manually confirmed through visual assessment of the MS/MS spectra to ensure their overall quality. In addition, a criterion for manual validation was applied, which required the presence of at least four readily observable y ions. Each sample underwent triplicate mass spectrometry analysis, and proteins identified in all three replicates in the experimental group but not in the control group were considered differentially expressed proteins.

The protein-protein interaction pathways were performed with String 9.1 Web software (SIB Swiss Institute of Bioinformatics, Lausanne, Switzerland).

### 4.4. Western Blotting

Western blots were performed using standard procedures. Protein extracts were electrophoresed through a precast gel (NuPAGE^®^Novex^®^ 4–12% Bis-Tris Gel, 1.5 mm, 10 wells, Invitrogen™, Carlsbad, CA, USA). Proteins were transferred from the gel to a polyvinyldifluoride (PVDF) membrane by means of the semidry technique using a Criterion Blotter (Bio-Rad, Hercules, CA, USA) at 100 V for 60 min and blocked with 5% milk in PBS (adjusted to pH 7.4) containing 0.05% Tween-20. The membranes were then separately incubated overnight with primary antibodies. After washing, membranes were incubated with HRP-conjugated secondary antibodies for one hour. Proteins were detected with an enhanced chemiluminescent (ECL) system, and quantitative analysis of the western blotting was carried out using ImageQuant-TL-7.0 software.

### 4.5. ELISA Analysis

The concentrations of amyloid protein precursor (APP), β-amyloid, and tau proteins in neuroblastoma cells were measured using the ELISA method. Each cell lysate sample was analyzed for the concentrations of expectant and candidate proteins in duplicate, using commercially available enzyme-linked immunosorbent assay (ELISA) kits. The protein concentrations were tested using standard protocols as suggested by the manufacturer. The ELISA reader model was the Multiskan EX (Thermo Fisher Scientific, Vantaa, Finland).

### 4.6. Statistical Analysis

All calculations were performed using SigmaStat v4.0 statistical software (Jandel Science Corp., San Rafael, CA, USA). All statistical significances were evaluated at a 95% confidence level or better. Data are presented as mean ± standard error. Statistical significance was determined using the Student’s *t* test at *p* < 0.05.

## 5. Conclusions

Overall, the findings of this study demonstrate that treatment with angiotensin-converting enzyme inhibitors promotes the expression of calreticulin, low-density lipoprotein-receptor-related protein 1B, and 14-3-3 protein zeta/delta in neuroblastoma cells. Calreticulin acts as a binding partner of APP and LRP1, potentially playing a role in the clearance of β-amyloid. Low-density lipoprotein receptor-related protein 1B is a membrane protein that regulates cholesterol metabolism and cell proliferation, while 14-3-3 protein zeta/delta regulates the activity of many cell cycle proteins, thereby affecting cell proliferation and growth. Therefore, these results suggest that angiotensin-converting enzyme inhibitors have the potential to affect the growth and proliferation of neuroblastoma cells by regulating the expression of these proteins.

## Figures and Tables

**Table 1:** Proteins identified with high confidence levels (at least three unique peptide sequences matched) in this study.

Accession No. ^a^	Protein Name	MW (KDa)	Mascot Score	Match Queries	PI	Sequence Coverage	Peptide ^b^
P63104	14-3-3 protein zeta/delta	27,728	66	4	4.76	15%	R.NLLSVAYKNVVGARR.S K.SVTEQGAELSNEER.N R.SSWRVVSSIEQK.T + deamidated (NQ); 2 phospho (ST) K.SVTEQGAELSNEER.N + 2 phospho (ST)
P27797	Calreticulin	123,665	32	6	5.5	6%	K.NVLINKDIR.C + deamidated (NQ) K.DKGLQTSQDAR.F + 2 deamidated (NQ); phospho (ST) K.GQTLVVQFTVK.H + deamidated (NQ); Phospho (ST) K.IDNSQVESGSLEDDWDFLPPKK.I K.IDNSQVESGSLEDDWDFLPPKK.I + deamidated (NQ) K.SGTIFDNFLITNDEAYAEEFGNETWGVTK.A
Q9NZR2	Low-density lipoprotein-receptor-related protein 1B	515,159	26	23	5.09	6%	K.CIPVNLR.C + carbamidomethyl (C) K.SCEPASPTCSSR.E + carbamidomethyl (C); 3 Phospho (ST) R.TCLSNCTASQFR.C + carbamidomethyl (C); deamidated (NQ); 2 Phospho (ST) K.CSQVCEQHKHTVK.C + carboxymethyl (C); 2 deamidated (NQ); phospho (ST) R.EYICASDGCISASLK.C + carbamidomethyl (C); 2 phospho (ST); phospho (Y) K.SDEKLLYCENRSCR.R + 2 carbamidomethyl (C); deamidated (NQ); phospho (ST) K.DQDECAVYGTCSQTCR.N + carbamidomethyl (C); carboxymethyl (C); phospho (ST) K.NCNNTDCTHFYKLGVK.T + carbamidomethyl (C); 3 deamidated (NQ); phospho (ST) K.DQDECAVYGTCSQTCR.N + carboxymethyl (C); 2 deamidated (NQ); 2 phospho (ST); phospho (Y) R.IIEVSKLNGLYPTILVSK.R + phospho (ST); phospho (Y) R.TNTLSKANKWTGQNVSVIQK.T + 2 deamidated (NQ); phospho (ST) K.CKSAEQSCNSSFFMCKNGR.C + carboxymethyl (C); 2 deamidated (NQ); 2 phospho (ST) K.CKSAEQSCNSSFFMCKNGR.C + carboxymethyl (C); 3 deamidated (NQ); Oxidation (M); 3 phospho (ST) K.LYWTDGNTINMANMDGSNSK.I + 2 deamidated (NQ); Oxidation (M); 3 phospho (ST) K.CKSAEQSCNSSFFMCKNGR.C + 2 carboxymethyl (C); 2 deamidated (NQ); Oxidation (M); 3 phospho (ST) R.GKLYWTDGNTINMANMDGSNSK.I + deamidated (NQ); 2 Oxidation (M); 3 phospho (ST) R.NTHGSYTCSCVEGYLMQPDNR.S + 2 carbamidomethyl (C); 2 deamidated (NQ); 3 phospho (ST) R.CIPKRWLCDGANDCGSNEDESNQTCTAR.T + carbamidomethyl (C); 2 deamidated (NQ); phospho (ST) R.NCHINECLSKKVSGCSQDCQDLPVSYK.C + 3 deamidated (NQ); 3 phospho (ST) K.SCEPASPTCSSREYICASDGCISASLK.C + carbamidomethyl (C); 2 carboxymethyl (C); 5 phospho (ST); phospho (Y) R.CNSTSLCVLPTWICDGSNDCGDYSDELK.C + carbamidomethyl (C); 5 phospho (ST) K.DDGKTCVDIDECSSGFPCSQQCINTYGTYK.C + carbamidomethyl (C); carboxymethyl (C); 3 deamidated (NQ); 5 phospho (ST); phospho (Y)

^a^ Swiss-Prot/TrEMBL accession number was obtained from http://us.expasy.org/; accessed on 28 April 2022. ^b^ Number of unique peptide sequences matched from Mascot database search result for this protein.

**Table 2:** Subcellular location and protein function of proteins with high confidence levels identified in neuroblastoma cells.

Protein Name	Subcellular Location	Biological Process	Molecular Function	Protein Function
14-3-3 protein zeta/delta	Cytoplasm	Cytoplasmic sequestering of protein	Histone deacetylase binding	Adapter protein implicated in the regulation of a large spectrum of both general and specialized signaling pathways. Binds to a large number of partners, usually by recognition of a phosphoserine or phosphothreonine motif. Binding generally results in the modulation of the activity of the binding partner. Negative regulator of osteogenesis. Blocks the nuclear translocation of the phosphorylated form (by AKT1) of SRPK2 and antagonizes its stimulatory effect on cyclin D1 expression resulting in blockage of neuronal apoptosis elicited by SRPK2
Calreticulin	Endoplasmic reticulum lumen	Cellular senescence	DNA binding	Calcium-binding chaperone that promotes folding, oligomeric assembly and quality control in the endoplasmic reticulum (ER) via the calreticulin/calnexin cycle. This lectin interacts transiently with almost all of the monoglucosylated glycoproteins that are synthesized in the ER. Interacts with the DNA-binding domain of NR3C1 and mediates its nuclear export. Involved in maternal gene expression regulation. May participate in oocyte maturation via the regulation of calcium homeostasis
Low-density lipoprotein-receptor-related protein 1B	Membrane	Protein transport	Calcium ion binding	Potential cell surface proteins that bind and internalize ligands in the process of receptor-mediated endocytosis.

## Data Availability

Not applicable.
